# Identification of Liver Epithelial Cell-derived Ig Expression in μ chain-deficient mice

**DOI:** 10.1038/srep23669

**Published:** 2016-03-29

**Authors:** Wenwei Shao, Chi Zhang, Enyang Liu, Long Zhang, Junfan Ma, Zhu Zhu, Xiaoting Gong, Zhihai Qin, Xiaoyan Qiu

**Affiliations:** 1 Department of Immunology, School of Basic Medical Sciences, Peking University Health Science Center, Beijing, 100191, China; 2 Key Laboratory of Medical Immunology, Ministry of Health, Beijing, 100191, China; 3 Department of Immunology, Institute of Biophysics Chinese Academy of Sciences, Beijing, 100101, China

## Abstract

Growing evidence indicates that B cells are not the only source of immunoglobulin (Ig). To investigate this discovery further, we used μMT mice, which have a disruption of the first transmembrane exon of the μ heavy chain and do not express the membrane form of IgM. These mice lack mature B cells and thus serve as a good model to explore Ig expression by liver epithelial cells. We found that Ig heavy chains (μ, δ, γ and α) and light chains (κ and λ) were expressed in sorted liver epithelial cells of μMT mice. Surprisingly, each heavy chain class showed its respective variable region sequence characteristics in their variable region, instead of sharing the same VDJ usage, which suggests that class switching does not occur in liver epithelial cells. Moreover, the γ and α chains, but not the μ and δ chains, showed mutations in the variable region, thus indicating that different classes of Ig have different activities. Our findings support the concept that non-B cells, liver epithelial cells here, can produce different classes of Ig.

Immunoglobulin (Ig) is one of the classic immune molecules and plays an important role in the body’s immune response. Currently, B lineage lymphocytes are considered to be the only source of Ig. However, since 20 years ago, a series of studies by our group proved that many non-B cancer cells, especially epithelial cancer cells, can also express Ig, including IgG, IgA and IgM^1^,^2^,^3^,^4^,^5^,^6^,^7^,^8^,^9^. Moreover, epithelial cancer cell-expressed IgG showed growth factor-like activity, which can promote cancer progression^1^,^2^,^10^. Subsequently, these unusual findings were confirmed by other researchers^11^,^12^,^13^,^14^,^15^,^16^,^17^,^18^,^19^. Recently, several types of Ig, including IgG, IgA and IgM, have been found in normal non-B cells, including epithelial cells, germ cells, neurons, endothelial cells, and even monocytes^4^,^5^,^13^,^20^,^21^. Moreover, normal epithelial cell-derived IgG, IgA and IgM showed characteristic antibody activity^9^,^22^. All of these studies have challenged the classical concept that B cells are the only source of Ig.

The functional membrane form of the IgM heavy chain (μ) is thought to be essential for B cell differentiation. B cells that lack μ chains are eliminated by the body^23^. The B cell-deficient μMT mice contain a disruption of the first transmembrane exon of the μ heavy chain and thus do not express the membrane form of IgM. These mice lack mature B cells due to a developmental block at the pro-B cell stage, after which B cells undergo apoptosis^24^. μMT mice are considered to be a suitable model to explore infection or tumor immunity in the absence of B cells and Ig production due to the lack of B cells^25^,^26^,^27^,^28^,^29^,^30^. However, growing studies have found that μMT mice contain both Ig, including IgA, IgG and IgE, and Ig-producing cells^31^,^32^,^33^,^34^. With regard to our previous finding that Ig can be found in many non-B cells in human and mice, we hypothesized that the Ig in μMT mice is mainly produced by non-B cells instead of the residual a small population of B cells as described above.

In this study, B cell-deficient μMT mice were used as a model to verify our hypothesis. We first identified IgM and IgA expression in several non-immune tissues, including liver, lung and kidney. The levels of IgM and IgA in these tissues were similar to those found in wild type (WT) mice, whereas the levels of serum Ig in the μMT mice were much lower than those in WT mice. Subsequently, we analyzed IgM, IgG, IgA, and IgD heavy and light chain transcripts and protein in sorted liver epithelial cells and found that liver epithelial cells could express different classes of Ig. Moreover, the liver epithelial cell-derived Ig transcripts displayed distinct characteristics compared with B cell-derived Ig transcripts.

## Results

### No mature B cells and low levels of serum Ig were detected in μMT mice

**Figure 1:**
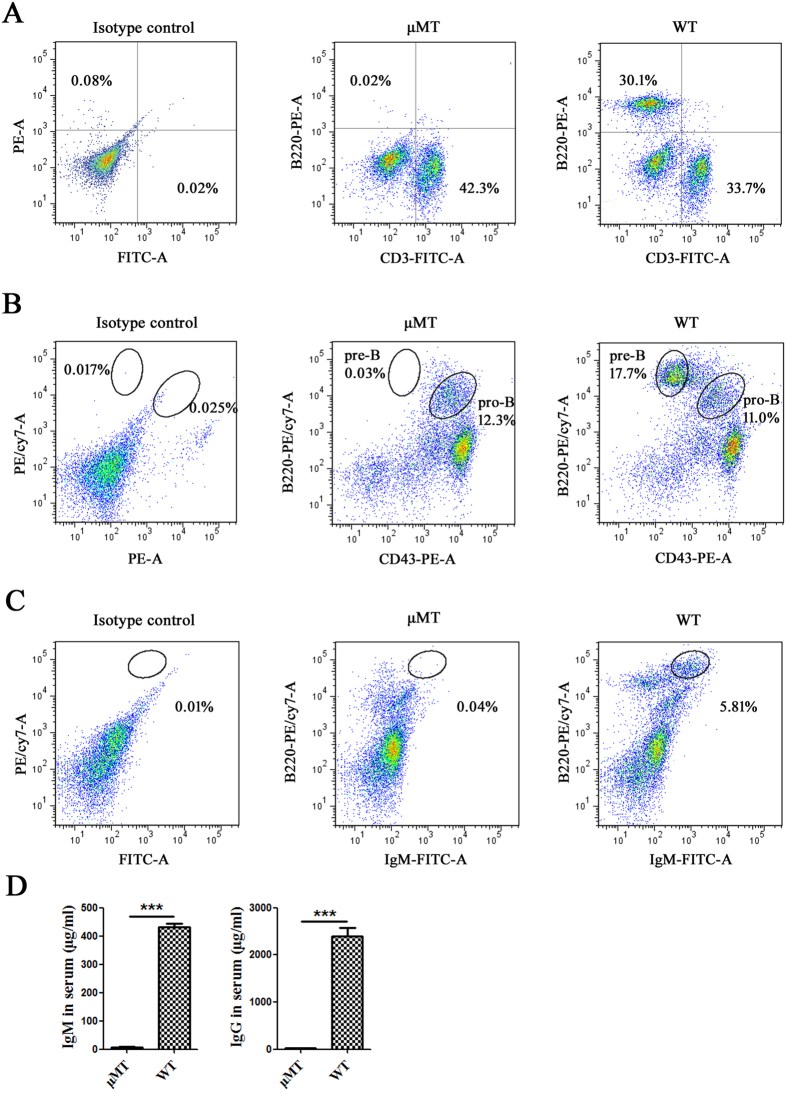
Identification of B cells and Igs in μMT mice. (**A**) B lymphocyte and T lymphocyte in peripheral blood of WT mice and μMT mice were detected with PE anti-mouse B220 and FITC anti-mouse CD3 by FACS. (**B**) Pro-B cell (CD43^+^ B220^low^) and pre-B cell (CD43^−^ B220^+^) in BM of WT mice and μMT mice were detected with PE anti-mouse CD43 and PE/Cy7 anti-mouse B220 by FACS. The cells in the upper left circle were pre-B cells, and the cells in the bottom right circle were pro-B cells. (**C**) Mature B cell (B220^+^ IgM^+^) in BM of WT mice and μMT mice were detected with FITC anti-mouse IgM and PE/Cy7 anti-mouse B220 by FACS. The circle showed mature B cells. (**D**) The concentrations of IgM and IgG in serum from WT mice and μMT mice were detected by ELISA. ***P < 0.001, **P < 0.01, ns P > 0.05.

We first detected whether there was residual mature B cells in μMT mice. In peripheral blood, there were no cells that stained with the B220 B cell marker (Fig. 1A). We then analyzed B cell development in the bone marrow (BM) of μMT mice. Unlike their WT counterparts (BALB/c mice), μMT mice contained neither pre-B cells (CD43^−^B220^+^) nor mature B cells (B220^+^ IgM^+^) in the BM (Fig. 1B,C). B cell development from pro-B to pre-B is prevented in μMT mice. Therefore, we used μMT mice as a B cell-deficient model.

Next, we measured the concentrations of IgM and IgG in the serum of μMT mice by ELISA, using the serum of WT mice as a control. The serum IgM and IgG levels in the μMT mice were reduced nearly 100-fold compared with WT mice (Fig. 1D).

### Detection of IgM and Igk in non-B cells in multiple tissues of μMT mice by immunostaining

**Figure 2:**
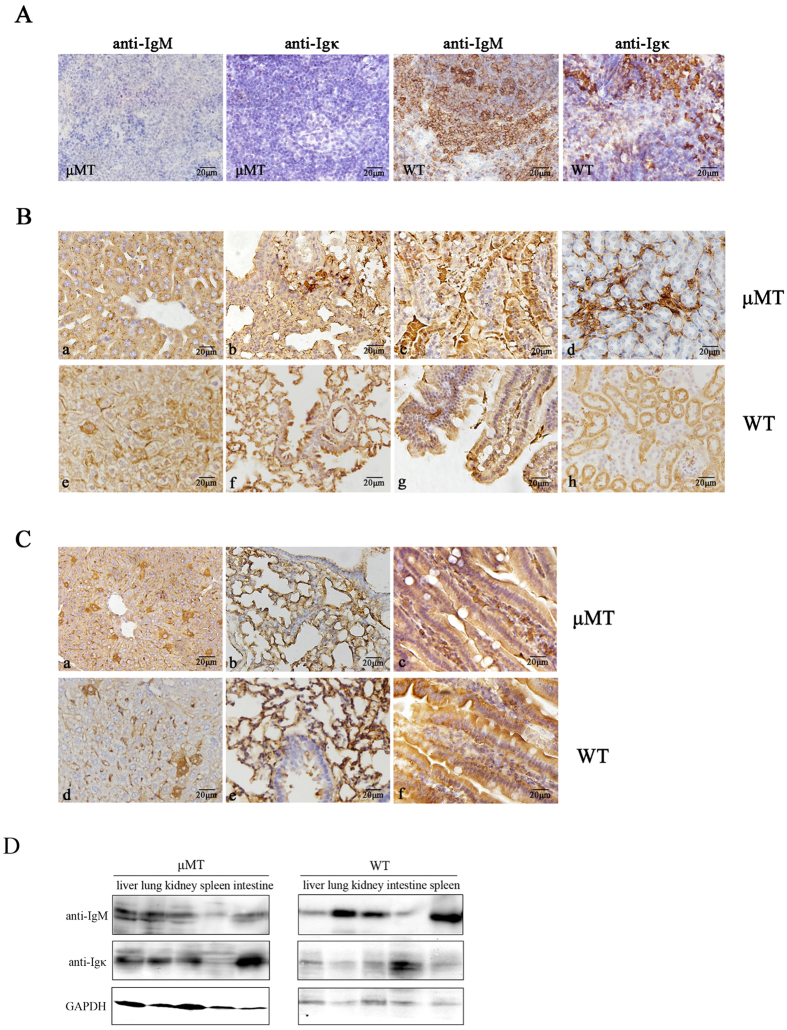
IgM and Igκ immunostaining in non B cells in multiple tissues of μMT mice by immunohisto-chemistry. (**A**) IgM and Igκ were detected in spleen of μMT mice and WT mice with anti-mouse IgM or Igκ antibodies by immunohistochemistry. (**B**) IgM was detected in liver (a,e), lung (b,f), small intestine (c,g) and kidney (d,h) of μMT mice and WT mice with goat anti-mouse IgM antibodies by immunohistochemistry. (**C**) Igκ was detected in liver (a,d), lung (b,e) and small intestine (c,f) of μMT mice and WT mice with goat anti-mouse Igκ antibodies by immunohistochemistry. (**D**) IgM and Ig κ were detected in tissues of μMT mice and WT mice with goat anti-mouse IgM or Igκ antibodies by western blot.

To determine whether Ig widely present in non-B cells of the μMT mice, we performed immunohistochemistry using anti-mouse IgM and anti-mouse Igκ chain antibodies. Although there was hardly any positive staining in the spleen (Fig. 2A), non-B cells (especially epithelial cells) in multiple tissues, including liver, lung, kidney, and small intestine, displayed a positive staining pattern that was equivalent to WT mice (Fig. 2B,C). In addition, the fact that Ig was widely present in non-B cells was confirmed by Western blotting (Fig. 2D).

### Different classes of Ig were found in livers of μMT mice

**Figure 3:**
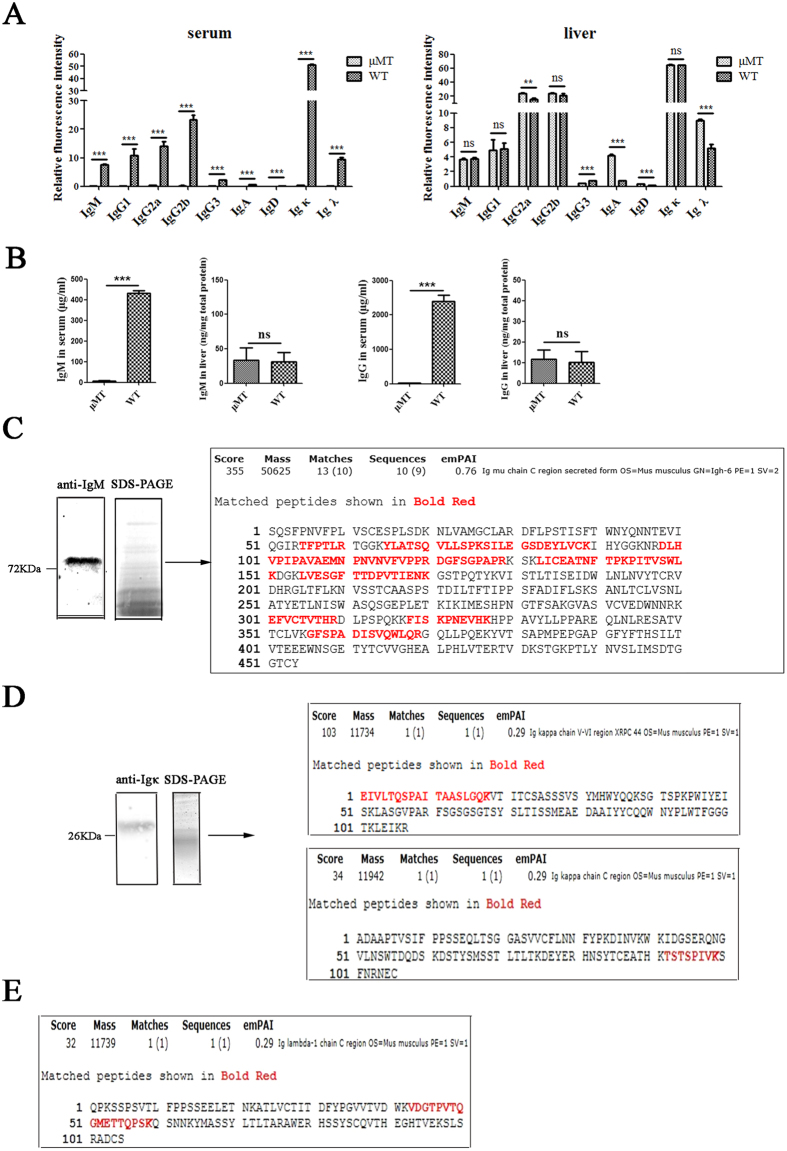
IgM, IgG, IgD, IgA, Ig κ and Igλ detection in serum and liver cells. (**A**) Ig expression level in serum and liver lysate of WT mice and μMT mice were measured by Mouse Ig Isotyping Array. WT serum was diluted 100 times, while serum from μMT mice was diluted 50 times. The statistical analysis was performed under the diluted condition of serum and the dilution difference was not taken into account. Igs were detected in 30 μg liver protein from WT mice or μMT mice. (**B**) The concentrations of IgM in serum and liver lysate from WT mice and μMT mice were detected by ELISA. ***P < 0.001, **P < 0.01, ns P > 0.05. (**C**) Liver lysate from μMT mice were separated by Superdex 200 and SDS-PAGE. Igκ was detected by goat anti-mouse Igκ antibody. Mass spectrometry analysis of a ~75 kDa band, which can be detected by Western blot, revealed several peptide that match mice Igμ heavy chain protein. (**D**) Liver lysate from μMT mice were separated by Superdex 200 and SDS-PAGE. Igκ was detected by goat anti-mouse Igκ antibody. Mass spectrometry analysis of a ~26 kDa band, which can be detected by Western blot, revealed several peptide that match mice Igκ light chain. (**E**) One peptide of Igλ light chain protein was detected in the mass spectrometry analysis of a ~26 kDa band.

To further explore the expression of Ig, we analyzed different classes of Ig in livers of μMT mice using a Rapid Mouse Ig Isotyping Array. First, blood was drawn from the eye canthus to obtain serum, and then the mice were perfused to remove blood cells and serum Ig. Protein was extracted from the liver, and the Ig in the liver lysate and the serum were quantitated by fluorescence with Rapid Mouse Ig Isotyping Array. As expected, there were low levels of Ig in the serum of μMT mice compared with WT mice. However, the levels of IgM, IgG1, IgG2b and Igκ in the μMT liver lysate were the same as those in the WT liver lysate. Moreover, the levels of IgA, IgD, Igλ and IgG2a were much higher in the μMT liver lysate than in the WT liver lysate (Fig. 3A). We also confirmed this characteristic of IgM and IgG in the liver lysate by ELISA (Fig. 3B).

Subsequently, to confirm that liver cells of μMT mice could produce Ig, we analyzed the μ and κ chains in the μMT liver lysate by mass spectrometry (MS). An SDS-PAGE band of liver lysate of approximately 75 kDa, which was stained with a goat anti-mouse IgM antibody by Western blotting, contained 11 peptides that correspond to the mice Ig μ heavy chain (Fig. 3C). Similarly, a band of approximately 26 kDa, which was detected by an anti-mouse Ig κ antibody by Western blotting, yielded 1 peptide that corresponded to a κ chain variable region and 1 peptide that corresponded to a κ chain constant region (Fig. 3D). In addition, 1 peptide corresponding to the λ chain constant region was found in the 26 kDa band (Fig. 3E).

### IgM and IgG were secreted by liver epithelial cells in μMT mice

**Figure 4:**
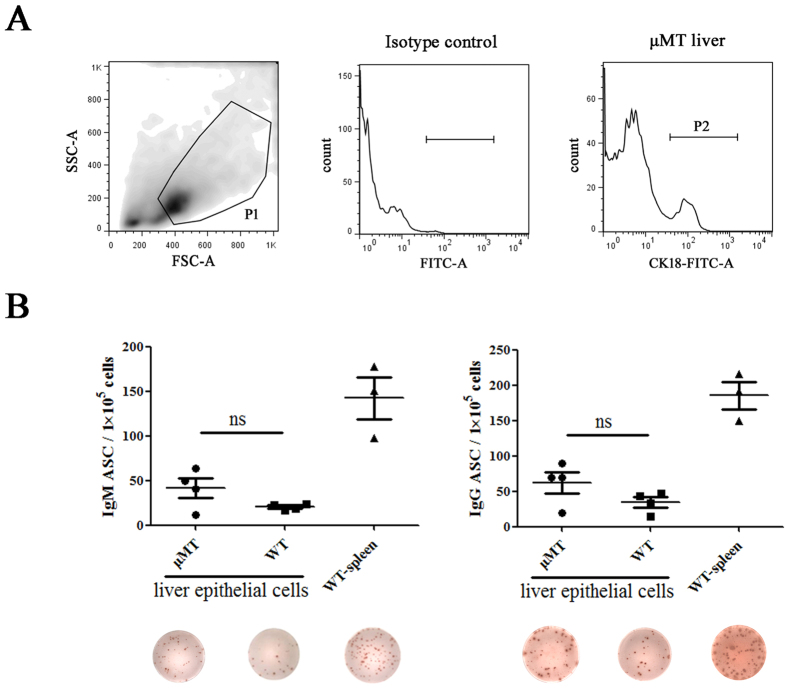
IgM and IgG were secreted from sorted liver epithelial cells. (**A**) CK18 was chosen as the sorting marker for liver epithelial cells. P1 represented the larger cells in liver. CK18^+^ cells in P1 gate, which were P2 cells, were harvested. (**B**) Secreted IgM and IgG of liver epithelial cells sorted by CK18 from WT mice and μMT mice were detected by ELISPOT. WT spleen cells were used as positive control.

To verify that the Ig found in the liver was produced by epithelial cells, we performed flow cytometric sorting of liver epithelial cells using the epithelial marker CK18 (Fig. 4A). We cultured the sorted cells and used ELISPOT to determine whether IgM or IgG was being secreted. In fact, sorted liver epithelial cells from both μMT and WT mice could secret IgM and IgG (Fig. 4B). Moreover, the levels of IgM and IgG were comparable between μMT and WT mice (Fig. 4B).

### Ig heavy and light chain transcripts in sorted liver epithelial cells of μMT mice

**Figure 5:**
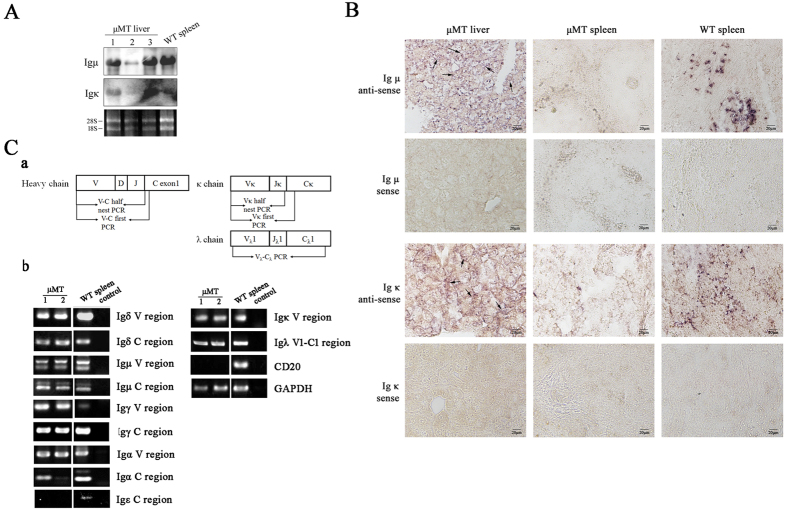
Ig gene rearrangement and transcription in μMT mice liver epithelial cells. (**A**) Northern blot analysis. Total RNA from liver cells of three μMT mice and WT spleen cells (positive control) were transferred to nylon membrane, and probed with probes to constant region of Igμ and Igκ; 28 s and 18 s RNA were shown by DNA agarose gel electrophoresis. (**B**) *In situ* hybridization analysis was shown for detection of Ig μ and Ig κ transcripts in μMT mice with antisense or sense probes for C region of μ chain or J-C region of κ chain. (**C**) a It was shown for the primer design of Ig μ variable region, Ig κ variable region and Igλ; b Sorted liver epithelial cells from two μMT mice were analyzed for Ig rearrangement and transcription by RT-PCR. WT spleen cells were used as the positive control. Variable region and constant region of heavy chain (μ, α, δ, γ, ε) and light chain (κ, λ), CD20 and GAPDH were amplified; “1, 2” represented two samples of sorted μMT liver epithelial cells.

To confirm the presence of μ and κ chain transcripts in the liver cells of μMT mice, we performed Northern blot analysis used the DNA fragments of Igμ or Igκ constant region as probes, the Igμ and κ chain transcripts were significantly found in liver tissues of μMT mice (Fig. 5A). Subsequently, the Igμ and κ chain transcripts were also determined in the liver epithelial cells of μMT mice with digoxigenin (DIG)-Labeled RNA fragments of Igμ CH3-CH4, or Jk-Ck as probes by *in situ* hybridization (Fig. 5B).

We used RT-PCR to analyze the expression of Ig in the sorted liver epithelial cells. The variable and constant regions of the different classes of heavy chains and light chains were amplified using a series of primers (Table S1). We detected rearranged transcripts of heavy chains (μ, δ, γ, and α) and light chains (κ and λ) in cDNA prepared from the sorted liver epithelial cells of μMT mice (Fig. 5C). However, we did not detect transcripts of the Ig ε chain. We also did not detect transcripts of CD20, a B cell marker, which confirmed that there was no B cell contamination in the cDNA libraries. The identities of all of the PCR products were confirmed by DNA sequencing, and these sequences have been submitted to the GenBank database (GenBank Accession Numbers: KT726173–KT726219).

### Ig variable region in sorted liver epithelial cells of μMT mice displayed distinct sequence characteristics

**Figure 6:**
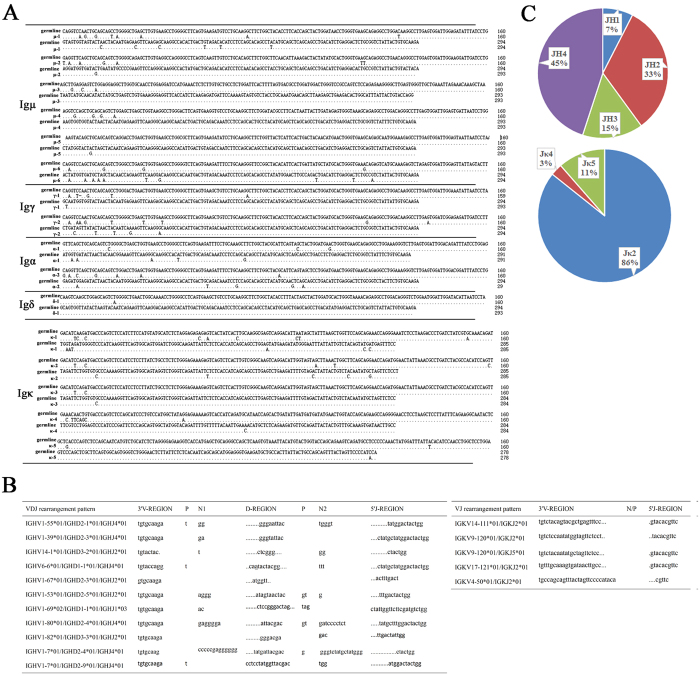
Characters of liver epithelial cell-derived Ig transcripts in μMT mice. (**A**) The mutation points of VDJ recombines and VJ recombines were shown, which were preponderant rearrangement pattern in μMT mice. “.” represents the matched nucleotides of liver epithelial cell-derived Ig transcripts compared with the Ig germline genes. (**B**) The V-D, D-J and V-J junctions of dominant liver epithelial cell-derived Igμ transcripts were shown; “.” represents the missing nucleotides of junctions compared with the Ig germline genes. Each point represents one nucleotide. (**C**) The frequency of JH and Jκ used by Ig derived from liver epithelial cells of μMT mice.

We cloned 86 VDJ rearrangements (from the heavy chain variable region) and 35 VκJκ rearrangements (from the κ chain variable region) that were expressed by the sorted liver epithelial cells from 5 μMT mice. Sequence analysis showed that 82 of the 86 VDJ rearrangements and all 35 VκJκ rearrangements were products of functional Ig rearrangements (Table 1). The analysis of Ig repertoires showed that similar to our previous findings^6^, in the same sorted epithelial cell population, the V, D, and J usage and VDJ sequences were dissimilar among the μ, γ, α and δ heavy chains. In fact, each class of heavy chain showed its own unique VDJ pattern (Table 1). The results further suggested that the liver epithelial cells did not possess a class switching mechanism. Interestingly, the μ, γ and κ transcripts showed a restricted clonal VDJ pattern, whereas the α and δ transcripts displayed VDJ diversity patterns similar to those exhibited by B cell-derived Ig and distinguished from Igμ and Igγ in liver epithelial cells (Table 1). To exclude an influence of the primers, we also amplified variable regions of the μ and κ chains from WT mice spleen cells and found the rearrangement patterns to be diverse (Table 2). In addition, we analyzed mutations in the representative VDJ and VκJκ rearrangements (Fig. 6A). The primers for the λ light chain were designed specific to V_λ_1/J_λ_1, and the products were confirmed to be V_λ_1/J_λ_1 by DNA sequencing (data not shown).

N/P addition in the V-D, D-J and V-J junctions is a classical feature of B cell-derived Ig, so we analyzed N/P addition in Ig transcripts derived from the liver epithelial cells. Markedly, 93% of the VDJ rearrangements had classical N/P addition, whereas only 5% of the VκJκ rearrangements had N/P addition (Fig. 6B). Interestingly, we found that 86% of the VκJκ rearrangements used Jκ2 and that VDJ rearrangements frequently used JH4 and JH2 in Ig expressed by the liver epithelial cells of μMT mice (Fig. 6C).

## Discussion

In this study, by using B cell-deficient μMT mice, we further provided evidence that Ig can be expressed by non-B cells under physiological conditions. We found that liver epithelial cells produced multiple classes of Ig, including IgG, IgM, IgA and IgD. These liver epithelial cell-derived Ig had distinctive characteristics in their variable regions.

In past 10 years, many studies have shown that non-B cell cancer cells, including epithelial cancer cells, and other types of malignant cells can produce Ig, especially IgG, which is involved in cell survival, proliferation, tumorigenesis and metastasis^35^,^36^,^37^,^38^,^39^. Ig expression in non-B cells was thought to be unique to malignant cells due to the genomic instability in these cells. However, in recent years, growing evidence has indicated that normal non-B cells, such as epithelial cells, endothelial cells, neurons, and even germ cells, can also express Ig. The expression of Ig in these non-B cells is of unknown significance, and its function remains poorly understood. Because these findings challenge the important classical theory that B cells are the only source of Ig, some immunologists are still questioning the possibility that non-B cells produce Ig. To address this issue, we avoided the contamination of B cells by using μMT mice (which have a deletion in the transmembrane domain of the Ig μ chain, thus blocking B cell development after the pro-B stage) as a model to determine whether Ig can be produced in non-B cells.

First, we found that mature B cells are not present in peripheral blood and lymphoid organs of μMT mice; furthermore, these mice have very low levels (~100 times lower than WT mice) of Ig in the bloodstream. Subsequently, using a series of techniques, including immunohistochemistry, western blotting, ELISPOT, MS, RT-PCR and DNA sequencing, we found that different classes of Ig could be expressed and secreted from non-B cells (such as liver epithelial cells) at both the mRNA and protein level. Moreover, the levels of Ig in non-immune organs in μMT mice was similar to those in WT mice, which suggests that non-B cells can spontaneously express Ig that is retained locally, whereas the Ig in the bloodstream may be derived mainly from B cells.

Hepatocytes are epithelial cells that are involved in synthesizing protein, cholesterol, bile salts, fibrinogen, phospholipids and glycoproteins. So far, there are no reports indicating that hepatocytes can express Ig or perform Ig-related functions. However, mouse liver tissue was used as a negative control by Tonegawa (1987 Nobel laureate in Physiology for discovery of Ig gene rearrangement) when he confirmed that Ig genes are rearranged in Ig-producing cells. In 1976, he used Southern blotting to show that a mouse myeloma cell line, but not liver tissue, was able to bind to a probe made from the mouse myeloma cell line’s mRNA fragment of an Ig variable region^40^,^41^,^42^. It was concluded that Ig gene rearrangement occurred in the myeloma cells but not in liver tissue. This conclusion further strengthened the classical concept that only B cells can produce Ig. However, in the current study, we found that Ig gene rearrangement also occurs in μMT mouse liver epithelial cells sorted by FACS with the specific liver epithelial cell marker CK18. In light of the current knowledge of Ig gene rearrangement, it is likely that the conclusion made by Tonegawa did not take substantial differences in Ig gene rearrangement among Ig-producing cells into account. Different conclusion could have been reached if Tonegawa had used a liver cell-derived Ig variable region mRNA fragment as a probe in his experiments. In addition, our results reveal a novel Ig source in μMT mice because Ig can be produced from many non-B cells, such as liver epithelial cells, but is not limited to z “a small population of B cells” as described previously^32^,^33^,^34^. In fact, we did not find any B cells in peripheral blood or mature B cells in BM in the μMT mice we used.

V(D)J gene rearrangement and transcription provide crucial evidence for Ig expression. We detected rearranged transcripts of Ig genes in sorted liver epithelial cells. To exclude the possible contamination of any other types of cells, we gated on the larger liver cells when CK18^+^ liver cells were sorted. As expected, rearranged functional heavy chain and light chain transcripts of IgG, IgM, IgD and IgA, but not IgE, have been found in the sorted liver epithelial cells. Sequencing analysis revealed that similar to B cells, the liver epithelial cells derived V(D)J or VJ showed classical Ig gene rearrangement patterns and had typical N/P additions among the V, D and J segments. Moreover, liver epithelial cell-derived IgA and IgG, but not IgM and IgD, also tended to show hypermutation. However, in the same liver epithelial cell population of μMT mice, each Ig heavy chain, including μ, δ, γ, α, showed distinctive variable region sequences, but not the same VDJ sequences. This finding suggests that class switching does not occur in liver epithelial cells, unlike the classical concept of class switching when IgM switches to IgA, IgG or IgE, only the constant region (not the variable region containing the VDJ sequences) is replaced. Indeed, we also found that non-B cells lack class switch mechanisms in our previous studies^6^. This finding suggests that the mechanism of Ig production in non-B cells is different from that in B cells, although the mechanism remains unknown.

Based on our current and previous results, non-B cell are able to express Ig; however, its function remains unclear. Our previous studies suggested that cervical cancer cell-produced IgM have natural IgM activity that could bind to ssDNA, dsDNA and bacteria^22^. Jiang *et al.* found that normal epidermal cells can express and secrete IgG and IgA, which can bind to different strains of bacteria^9^. We have preliminary results showing that IgM extracted from liver tissue of both WT and μMT mice possesses natural IgM activity (data not shown). This finding suggests that Ig from non-B cells, especially epithelial cells, may serve natural antibody functions and may be involved in natural immunity in local tissues. Our focus will be on the function of these Ig.

## Materials and Methods

### Mice

BALB/c mice were purchased from Charles River Laboratories (Beijing, China). μMT/BALB/c mice were housed in Institute of Biophysics, Chinese Academy of Science. All these mice used in this study were housed in a specific pathogen free environment at the Peking University Health Science Center. And procedures about animal studies were performed in accordance with the guidelines of the People’s Republic of China Ministry of Health and approved by the Animal Care and Use Committee of Peking University Health Science Center.

### Immunohistochemistry

At first, tissues were sectioned and fixed in 10% formalin, and then was embedded in paraffin. The paraffin sections were rehydrated by ethanol with the concentrations of 75%, 80%, 90%, 95% and 100% after deparaffinization. Antigen retrieval was performed in tris-EDTA buffer (pH 9.0) at 90 °C for 5-min in a microwave oven. To block the andogenous peroxidase activity, the slide was treated with 0.3% hydrogen peroxide for 5 min, and washed in PBS. Then, the sections were blocked with 10% normal goat serum for 10 min. Slides were incubated with primary antibody, goat anti-mouse IgM (VECTOR LAB, CA, USA) or goat anti-mouse Igκ (Southern Biotech, UBA, USA), for 60 min at 37 °C in a humidified chamber. After washed, the sections were incubated with the HRP-conjugated second antibodies (rabbit anti-goat IgG) at 37 °C for 40 min. After washing with PBS, the signal was detected using DAB (Dako, CA, USA). Sections stained without primary antibody were used as negative controls.

### Protein extraction

Heart perfusion was performed to the mice anaesthetized by chloralic hydras before sacrification. The tissues were cut into small pieces and lysed in TSD buffer (50 mM Tris-HCL, 0.5% sodium dodecyl sulfate, 5 mM DTT) followed by ultrasonication. Lysate were centrifuged at 13,000 rpm for 20 min at 4 °C, and the supernatants were collected for ELISA or Western blot.

### Rapid Mouse Ig Isotyping Array

As mentioned above, tissues lysate was prepared. Serum from mice was diluted for 100 times. Then the Ig was detected according to the instructions of Rapid Mouse Ig Isotyping Array (Ray Biotech, Norcross GA, USA).

### ELISA

To detect the concentration of IgM and IgG in liver lysate of mice liver and serum, we used Mouse IgM ELISA Quantitation Set and Mouse IgG ELISA Quantitation Set (Bethyl Laboratories, USA). 80 μg liver lysate or 500 time diluted serum was performed ELISA analysis by following the standard protocol. OD450 was measured with a microplate reader (Bio-Tek, Winooski, VT).

### SDS-PAGE, Western Blot and MS

The reduced (with ß-mercaptoethanol) tissue lysate samples were separated by SDS-PAGE. For Western blot, the separate proteins were transferred to nitrocellulose membranes. After blocked with tris-buffered saline containing and 5% defatted milk for 2 h at room temperature, membranes were incubated with the appropriate primary antibodies: Biotin-conjugated goat anti-mouse IgM (μ chain specific) antibody (VECTOR LAB, CA, USA) and goat anti-mouse Ig κ antibody (Southern Biotech, UBA, USA) overnight at 4 °C. Following the wash with TBS, membranes were incubated with the appropriate IRDye 800 or IRDye 700-conjugated secondary antibodies (LI-COR Bioscience Inc., Lincoln, NE, USA) in the dark. The Odyssey Imaging System (LI-COR Bioscience Inc., Lincoln, NE, USA) was used to detect the signals. For MS, the SDS-PAGE bands at specific molecular weight were cut to perform MS analysis.

### Isolation of mice liver epithelial cells

Heart perfusion was performed to the mice anaesthetized by chloralic hydras before sacridication. The livers were cut into little pieces and digested with 150 U/ml DNase I (Sigma, St. Louis, Mo, USA) and 500 U/ml collagenase IV (Sigma, St. Louis, Mo, USA) at 37 °C. Then the liver single-cell suspensions was obtained after filtering by mesh. The liver cells were stained with rabbit anti-mouse CK 18 (Santa Cruz, San Diego, CA, USA), and then were further labeled with secondary antibody (FITC-conjugated anti-rabbit IgG). The CK18^+^ liver epithelial cells were sorted by FACSAria™ II Flow Cytometer (Becton Dickinson, San Diego, CA, USA). The sorted liver epithelial cells were used for RT-PCR, ELISPOT and WB analysis.

### Flow cytometry

To analyze B cells in mice, blood cells and bone marrow cells were harvest and washed with PBS. Before staining, cells were blocked with 5% FBS in PBS for 30 min at 4 °C. And then cells were stained with membrane marker antibodies: PE-CD43, FITC-IgM, PE-B220 and PE/cy7-B220 purchased from eBioscience (USA). The control was performed that cells were stained with corresponding fluorescein conjugated isotype control antibody.

### ELISPOT

To detect the secretion of IgG and IgM in liver epithelial cells, ELISPOT was performed. 1 × 10^5^ sorted liver epithelial cells or 1 × 10^5^ spleen cells were used for the IgM or IgG ELISPOT. MultiScreen filter plates for ELISPOT were coated with goat anti-mouse IgM (Bethyl Laboratories, USA) or goat anti-mouse IgG (Bethyl Laboratories, USA) for overnight at 4 °C. Following the blocking with 10% FBS, cells were incubate with plates for 24 h at 37 °C. After washed with PBS, HRP conjugated detection antibody were incubated for 1 h at room temperature. Then AEC coloration was performed and analyzed by ELISPOT reader.

### Northern blot analysis

Total RNA was extracted from liver or spleen cells with TRIzol reagent. A total of 10 μg liver-derived RNA or 5 μg spleen-derived RNA was separated in 1% agarose gel with formaldehyde, and then transferred to a nylon membrane. The sequence of probe specific to Igμ constant region gene was: ttcatctctgcgacagctggaatgggcacat, and the probe specific to Igκ constant region was: cgccattttgtcgttcactgccatcaatcttc. The probes were conjugated with biotin. The membrane was hybridized with 60 ng/ml denatured probe in hybridization buffer (Thermo) after prehybrization at 56 °C for 12–16 h. And the signal was detected with North2South^®^ Chemiluminescent Hybridization and Detection Kit (Thermo) according to the manufacturer’s instructions.

### *In situ* hybridization

*In situ* hybridization was performed on 6 μm serial sections of paraffin-embedded tissue sections. Plasmids inserted with constant region fragments of the Igμ and Igκ, which were obtain by PCR with Igμ CH3-CH4 primers and Jk-Ck primers (Table S1), were linearized. The RNA probes were labeled with digoxigenin (DIG) and transcribed by T7 RNA polymerase (for the antisense probe) or SP6 RNA polymerase (for the sense probe) by DIG northern starter kit (Roche, Rotkreuz, Switzerland). Paraffin-embedded tissue sections were performed deparaffinized and dehydrate. Then sections were treated with Proteinase K, fixed with paraformaldehyde, prehybridized at 42 °C for 2 h, and hybridized with the DIG-labeled RNA probe (5 μg/mL) at 42 °C overnight. After hybridization, the sections were washed in 2 × SSC and 0.1 × SSC at 37 °C, respectively, and then treated with RNase A. The samples were incubated with alkaline phosphatase-conjugated antidigoxigenin antibody (1:250; Roche, Rotkreuz, Switzerland). BCIP/NBT (Sigma, Saint Louis, USA) was used for the color reaction. Corresponding sense probes were used as controls.

### RNA extraction and RT-PCR

Total RNA in spleen was extracted using TRIzol reagent (Invitrogen, Carlsbad, USA). For the sorted liver epithelial cells, RNA was extracted with RNeasy Mini Kit (Qiagen, Hilden, Germany) according to the standard protocol. Reverse transcription (RT) was carried out with RevertAid First Strand cDNA Synthesis Kit (Fermentas, Glen Burnie, USA) by following the manufacturer’s instructions. And then the cDNA was prepared for PCR.

The primers used in this study were shown in Table S1. The Ig constant region genes were amplified at the annealing temperature of 56 °C for 35 cycles. Semi-nested PCR was performed for Ig V region genes. cDNA derived from BALB/c mice spleen cells was used as positive control. PCR products were separated with 1% agarose gel by electrophoresis, and then were stained with ethidium bromide. The PCR products were cloned to pGEM-T Easy Vector (Promega) to be sequenced by ABI 3730XL Genetic Analyzer.

### Sequence analysis

In order to analyze the usage and junctions of Ig variable region genes, all sequence derived from PCR products were submitted to the IMGT V-QUEST program. Alignments were performed with Lasergene software (DNAStar) or with BLAST in NCBI to compare with published sequences.

### Statistical analysis

All statistical calculations were performed using the statistical software program (GraphPad Prism 5.0 software). Differences between different groups were evaluated by the Student’s *t* test. Differences were considered to be statistically significance when P was <0.05.

## Additional Information

**How to cite this article**: Shao, W. *et al.* Identification of Liver Epithelial Cell-derived Ig Expression in µ chain-deficient mice. *Sci. Rep.* **6**, 23669; doi: 10.1038/srep23669 (2016).

## Supplementary Material

Supplementary Information

## Figures and Tables

**Table 1:** VDJ and VJ recombinations in liver epithelial cells of μMT mice.

	Igμ			Igα			Igγ			Igδ			Ig κ		
	V_H_DJ_H_ recombination	No. of clones	Identity with germline (%)	V_H_DJ_H_ recombination	No. of clones	Identity with germline (%)	V_H_DJ_H_ recombination	No. of clones	Identity with germline (%)	V_H_DJ_H_ recombination	No. of clones	Identity with germline (%)	VJ recombination	No. of clones	Identity with germline (%)
No. 1	IGHV1-55*01/IGHD2-1*01/IGHJ4*01	7/8	97	IGHV1-80*01/IGHD2-4*01/IGHJ4*01	5/9	97.6	IGHV1-53*01/IGHD2-5*01/IGHJ2*01	5/7	99.6	IGHV17*01/IGHD2-4*01/IGHJ4*01	3/8	99.6	IGKV14-111*01/IGKJ2*01	7/8	96
				IGHV1-72*01/IGHD2-1*01/IGHJ2*01	2/9	97.3				IGHV1-39*01/IGHD2-3*01/IGHJ2*01	2/8	100			
	IGHV1-37*01/IGHD2-13*01/IGHJ2*01	1/8	98.6	IGHV1-64*01/IGHD2-9*01/IGHJ2*01	1/9	98	IGHV1-39*01/IGHD2-1*01/IGHJ3*01	2/7	95.1	IGHV1-55*01/IGHD1-1*01/IGHJ3*01	1/8	100	IGKV9-120*01/IGKJ2*01	1/8	100
				IGHV7-1*02/IGHD2-1*01/IGHJ2*01	1/9	99.6				IGHV1-80*01/IGHD2-4*01/IGHJ4*01	1/8	100			
										IGHV1-18*01/IGHD2-3*01/IGHJ4*01	1/8	99.3			
No. 2	IGHV14-1*01/IGHD3-2*01/IGHJ2*01	4/6	98.6	IGHV1-82*01/IGHD3-3*01/IGHJ2*01	3/6	97	IGHV1-69*02/IGHD1-1*01/IGHJ1*03	5/7	97	IGHV1-7*01/IGHD2-9*01/IGHJ4*01	3/8	100	IGKV9-120*01/IGKJ2*01	5/8	97.5
				IGHV1-81*01/IGHD5-8*01/IGHJ4*01	1/6	97				IGHV1-76*01/IGHD2-4*01/IGHJ4*01	1/8	100			
	IGHV1-82*01/IGHD1-1*01/IGHJ1*03	1/6	95.2	IGHV1-82*01/IGHD2-4*01/IGHJ3*01	1/6	97.6				IGHV1-39*01/IGHD1-1*01/IGHJ4*01	1/8	100	IGKV9-120*01/IGKJ2*01	2/8	99.6
				IGHV1-18*01/IGHD2-5*01/IGHJ2*01	1/6	96.3	IGHV1-78*01/IGHD2-3*01/IGHJ3*01	2/7	97.9	IGHV1-20*01/IGHD2-3*01/IGHJ3*01	1/8	99.3			
	IGHV1-55*01/IGHD1-2*01/IGHJ4*01	1/6	97							IGHV1-4*01/IGHD2-1*01/IGHJ4*01	1/8	100	IGKV14-111*01/IGKJ2*01	1/8	98.9
										IGHV1-4*01/IGHD2-9*01/IGHJ4*01	1/8	99.3			
No. 3	IGHV6-6*01/IGHD1-2*01/IGHJ4*01	5/8	99.7	—			—			—			IGKV9-120*01/IGKJ5*01	2/4	99.6
	IGHV1S16*01/IGHD2-10*02/IGHJ2*01	1/8	96										IGKV9-120*01/IGKJ2*01	1/4	99.6
	IGHV1-47*01/IGHD1-1*01/IGHJ2*01	1/8	100										IGKV9-120*01/IGKJ4*02	1/4	99.6
	IGHV1-7*01/IGHD4-1*02/IGHJ2*01	1/8	100												
No. 4	IGHV1-39*01/IGHD2-3*01/IGHJ4*01	5/9	100	—			—			—			IGKV17-121*01/IGKJ2*01	6/8	99.6
	IGHV14-3*02/IGHD5-8*01/IGHJ3*01	4/9	100										IGKV17-121*01/IGKJ5*01	2/8	99.3
No. 5	IGHV1-67*01/IGHD2-3*01/IGHJ2*01	5/5	96	—			—			—			IGKV4-50*01/IGKJ2*01	5/7	99.6
													IGKV4-50*01/IGKJ5*01	2/7	99.6

**Table 2:** V(D)J recombination of Ig gene in WT spleen cells.

WT-Spleen	No. of clones	Recombination	V segment identity withgermline (%)
VμDJμ recombination	2/8	IGHV1-80*01/IGHD2-4*01/IGHJ3*01	100
	1/8	IGHV1-47*01/IGHD2-5*01/IGHJ4*01	97.6
	1/8	IGHV1-82*01/IGHD2-9*01/IGHJ1*03	100
	1/8	IGHV1-31*01/IGHD2-3*01/IGHJ3*01	99.2
	1/8	IGHV1-7*01/IGHD2-5*01/IGHJ2*01	99.2
	1/8	IGHV1-78*01/IGHD2-14*01/IGHJ1*03	98.8
	1/8	IGHV1-76*01/IGHD2-3*01/IGHJ2*01	100
VκJκ recombination	1/7	IGKV9-120*01/IGKJ2*01	99.6
	1/7	IGKV9-120*01/IGKJ2*01	99.3
	1/7	IGKV14-111*01/IGKJ2*01	100
	1/7	IGKV14-126*01/IGKJ2*01	100
	1/7	IGKV17-127*01/IGKJ2*01	100
	1/7	IGKV17-127*01/IGKJ2*01	97.9
	1/7	IGKV17-127*01/IGKJ5*01	100
