# Supplementary material for: Identification of Liver Epithelial Cell-derived Ig Expression in μ chain-deficient mice
Source: Sci Rep. 2016 Mar 29;6:23669. doi: 10.1038/srep23669 (PMC4810322; doi:10.1038/srep23669)
Supplement: Supplementary Information [file srep23669-s1.doc]

**Identification of Liver Epithelial Cell-derived Ig Expression in μ chain-deficient mice**

Wenwei Shao^1^, Chi Zhang^1^, Enyang Liu^2^, Long Zhang^2^, Junfan Ma^1^, Zhu Zhu^1^, Xiaoting Gong^1^, Zhihai Qin^3*^, Xiaoyan Qiu^1,2*^

^1^ Department of Immunology, School of Basic Medical Sciences, Peking University Health Science Center, Beijing, 100191, China

^2^ Key Laboratory of Medical Immunology, Ministry of Health, Beijing, 100191, China

^3^ Department of Immunology, Institute of Biophysics Chinese Academy of Sciences, Beijing, 100101, China

^*^Corresponding author: Xiaoyan Qiu, MD, PhD, Center for Human Disease Genomics, Peking University, 38 Xue-yuan Road, 100191, Beijing, China. Tel.: 86-10-82805477; Fax: 86-10-82801149; E-mail: [qiuxy@bjmu.edu.cn](mailto:qiuxy@bjmu.edu.cn).

Zhihai Qin, Institute of Biophysics, Chinese Academy of Sciences, 15 Datun Road, Beijing 100101, China. Phone: 86-10-64888435; Fax: 86-10-64888570; E-mail: [zhihai@ibp.ac.cn](mailto:zhihai@ibp.ac.cn)

**Table S1. The sequences of PCR primers used in this study**

| **Gene Name** |  | **Primer Sequence 5’-3’** | **Product Size (bp)** |
| --- | --- | --- | --- |
| Ig μ  C region | Sense primer | CCTGGCAACCTATGAAAC | 449 |
|  | Antisense primer | GGATGCTGTGGGTAAAGT |  |
| Ig μ  variable region | The same sense primer (MH1) | CTTCCGGAATTCSARGTNMAGCTGSAGSAGTC | 380 |
|  | The same sense primer (MH2) | CTTCCGGAATTCSARGTNMAGCTGSAGSAGTCWGG |  |
|  | External antisense primer | CTTCAAGAAGGTGAGACCC |  |
|  | Internal antisense primer | GGAAGATCTGACATTTGGGAAGGACTGACTCTC |  |
| Ig κ variable region | Sense primer | GACATTCAGCTGACCCAGTCTCCA | 311 |
|  | External antisense primer | GTCCTGATCAGTCCAACTGTTCA |  |
|  | Internal antisense primer | AGNTTGGTTCCACCYCCGAACG (Y=T/C) |  |
| Ig γ  C region | Sense primer | CTGGGATGCCTGGTCAAGGGCTA | 425 |
|  | Antisense primer | GTCTGAGCTGTGTGYACYTCCAC |  |
| Ig δ  C region | Sense primer | CCTCTCAGAGTGCAAAGCCC | 602 |
|  | Antisense primer | GTGATGAGCTCAGAGCGACT |  |
| Ig α  C region-1 | Sense primer | GTCTGCGAGAAATCCCACCA | 398 |
|  | Antisense primer | CATCTGAACCCAGGAGCAGG |  |
| Ig α  C region-2 | Sense primer | ACCGTAAACTTCCCACCTGC | 431 |
|  | Antisense primer | AATGATGCGCCACTGTTCCA |  |
| Ig ε  C region | Sense primer | GGCTACGGCTATCAGTGCAT | 494 |
|  | Antisense primer | GTGTTACCAAGGCTAGTGGA |  |
| Ig λ variable region | Sense primer | CAGGCTGTTGTGACTCAGGAATCT | 635 |
|  | Antisense primer | GCACGGGACAAACTCTTCTCCACA |  |
| Ig γ  variable region | The same sense primer (MH1) | SARGTNMAGCTGSAGSAGTC | 380 |
|  | The same sense primer (MH2) | SARGTNMAGCTGSAGSAGTCWGG |  |
|  | External antisense primer | CTTCAAGAAGGTGAGACCC |  |
|  | Internal antisense primer | GGAAGATCTGACATTTGGGAAGGACTGACTCTC |  |
| Ig δ  variable region | The same sense primer (MH1) | SARGTNMAGCTGSAGSAGTC | 380 |
|  | The same sense primer (MH2) | SARGTNMAGCTGSAGSAGTCWGG |  |
|  | External antisense primer | GCTTTGCACTCTGAGAGGAG |  |
|  | Internal antisense primer | CATGTCAGGTCCCTTTTCATTAC |  |
| Ig α  variable region | The same sense primer (MH1) | SARGTNMAGCTGSAGSAGTC | 700 |
|  | The same sense primer (MH2) | SARGTNMAGCTGSAGSAGTCWGG |  |
|  | External antisense primer | AATGATGCGCCACTGTTCCA |  |
|  | Internal antisense primer | AATGATGCGCCACTGTTCCA |  |
| CD20 | The same sense primer | TTCAAACTTCCAAGCCGTATG | 241 |
|  | External antisense primer | GAGTTTAAGGAGCGATCTC |  |
|  | Internal antisense primer | GACAGCAGAACCACATTAGAT |  |
|  | Antisense primer | GTGATGAGCTCAGAGCGACT |  |
